# Phase-Based Thermal Wave Analysis for Lateral Characterization of Subsurface Defects in Solid Materials via Modeling and Simulation

**DOI:** 10.3390/ma18163753

**Published:** 2025-08-11

**Authors:** Botao Ma, Chen Liu, Shupeng Sun, Lin Zhang

**Affiliations:** Department of Engineering Mechanics, School of Civil Engineering, Shandong University, Jinan 250061, China; 202214991@mail.sdu.edu.cn (B.M.); 202415030@mail.sdu.edu.cn (C.L.); shpsun@sdu.edu.cn (S.S.)

**Keywords:** thermal wave analysis, lock-in thermography, thermal diffusion length, nondestructive testing, subsurface defect detection, finite element simulation

## Abstract

Lock-in thermography is a widely adopted infrared nondestructive testing technique that detects subsurface defects by applying modulated thermal waves and analyzing the resulting surface temperature variations. However, quantitatively characterizing subsurface defects at varying depths remains a significant challenge. This study explores the lateral resolution of subsurface defect detection using phase-based lock-in thermography, integrating analytical modeling, finite element simulation, and phase difference analysis. The results demonstrate that defect visibility and boundary definition are highly influenced by the excitation frequency. The thermal diffusion length, which is inversely proportional to the square root of the excitation frequency, governs both the penetration depth and the lateral spread of thermal energy. Higher frequencies enhance lateral resolution, whereas lower frequencies improve the detectability of deeper defects. Detection becomes particularly difficult for defects with small radii or low radius-to-depth ratios. A critical radius-to-depth threshold of 2 is identified as essential for reliable boundary delineation. These findings offer practical guidance for selecting excitation frequencies to achieve an optimal balance between depth sensitivity and lateral resolution in thermal-wave-based nondestructive evaluation.

## 1. Introduction

Nondestructive testing (NDT) is essential in modern materials science and engineering, offering reliable evaluation of defects without impairing the structural integrity of components. By enabling early detection of cracks, voids, delaminations, and surface irregularities, NDT ensures material reliability, prolongs service life, and supports safety-critical applications across aerospace, automotive, and civil infrastructure [1,2,3]. Recently, infrared thermography (IRT) has emerged as a powerful nondestructive testing technique for detecting and characterizing subsurface defects in various materials [4,5,6]. When a material containing subsurface anomalies is subjected to thermal excitation, temperature gradients caused by these anomalies become visible on the surface and can be recorded through infrared radiation. By monitoring the temperature variations induced by internal anomalies, IRT enables the evaluation of hidden flaws without physically altering the material. IRT is generally classified into two modes: passive and active. Passive thermography relies on naturally occurring or ambient heat to reveal defects, which makes it suitable for large-scale applications such as predictive maintenance and structural health monitoring of buildings and roads [7,8]. However, it is often limited by sensitivity and spatial resolution. Active thermography overcomes these limitations by applying controlled thermal excitation to enhance the detectability of defects. Depending on the type of thermal stimulus, active methods include lock-in thermography (LIT), step heating, vibrothermography, and thermal wave radar thermography [5,9,10,11,12,13,14,15,16].

Among active thermography techniques, LIT stands out for its high sensitivity, phase-based imaging, and strong resistance to background thermal noise [17,18,19,20]. In LIT, a periodic heat source, which is typically sinusoidal or square-wave, is applied to the specimen, and an infrared camera captures the thermal response. The phase shift between the input excitation and the response provides information about subsurface features, with greater contrast at defect locations. This phase-based approach enables defect detection at varying depths and offers robustness against nonuniform heating and variations in emissivity [21,22,23,24]. The location and shape of defects are usually determined based on the phase difference map. Several post-processing methods, including principal component thermography and the Canny operator, have been employed to improve the detection accuracy of defects by phase maps [25,26,27,28].

Thermal diffusion length governs how far the thermal wave reaches in materials and is defined by $\mu =\sqrt{\alpha /\left(\pi f\right)}$, where α is the thermal diffusivity and *f* is the excitation frequency. Adjusting the excitation frequency allows LIT to probe different defect depths, making it suitable for detecting defects, such as delaminations, cracks, and corrosion in multilayered and composite structures used in aerospace and civil engineering [12,29]. Although the frequency of thermal excitation determines thermal penetration depth (thermal diffusion length in the depth direction), it is required to measure at multiple frequencies for depth analysis of the defects [30]. As a result, this process can be very time-consuming, especially for large and thick specimens. A practical solution is periodic thermal excitation with varying frequencies, such as square waves or chirps [29,31,32]. A low-duty-cycle square wave method was proposed to address this issue [33]. In data analysis, lock-in amplifiers or Fourier transforms can be used to extract information in the frequency domain.

Thermal diffusion length affects detection in both depth and lateral directions in LIT. With phase-difference maps at various frequencies, information such as the optimal and blind frequencies can be determined by the phase difference to the frequency curve at each point. The blind frequency was applied to quantitatively estimate the defect depth, calculated as 1.5 to 2.0 times the thermal diffusion length at the blind frequency for the material under investigation [34]. This principle has been validated across various materials, including thermal barrier coatings and fiber-reinforced composites [26,30]. On the other hand, lateral thermal conduction tends to blur defect boundaries [30], reducing detectability, especially for small or shallow defects. Meola et al. [35] investigated the effects of defect size, depth, and thickness in composite panels. They found that thin defects in composites were much more challenging than thicker defects. Maldague mentioned an empirical rule of thumb that for a homogeneous isotropic material, the minor detectable defect should be no deeper than its diameter [36]. Wallbrink demonstrated that both the depth and diameter of the defect influence the phase angle, and the minimum detectable diameter is limited to 3 and 11.5 mm for defect depths of 3 and 8 mm, respectively [37]. B.B. Lahiri reported that the defect cannot be detected when the ratio of defect size to depth is less than one [38]. Recently, deep learning has shown great promise in quantitative defect detection with infrared thermography [39,40,41,42,43]. In summary, LIT has significantly advanced the estimation of defect size and depth [44].

Although prior studies have qualitatively recognized the impact of thermal diffusion, a comprehensive understanding of how excitation frequency interacts with defect geometry to influence lateral detectability remains underdeveloped. Addressing this knowledge gap is crucial for advancing the quantitative capabilities of phase-based thermographic techniques. To address these challenges, this study investigates the influence of geometric and thermal parameters—including defect depth, thickness, lateral radius, and the radius-to-depth ratio—on phase-based detectability in LIT. An analytical model was developed to consider heat conduction only in the depth direction, whereas three-dimensional finite element simulations were employed to capture lateral heat conduction effects. The results reveal that lateral conduction critically limits boundary sharpness, and the radius-to-depth ratio serves as a key indicator for lateral detectability. These findings should provide frequency selection strategies and improve the quantitative accuracy of thermal-wave-based nondestructive evaluation methods.

## 2. Theoretical Models and Methods

### 2.1. One-Dimensional Model of Three Layers for Thermal Response Analysis

**Figure 1:**
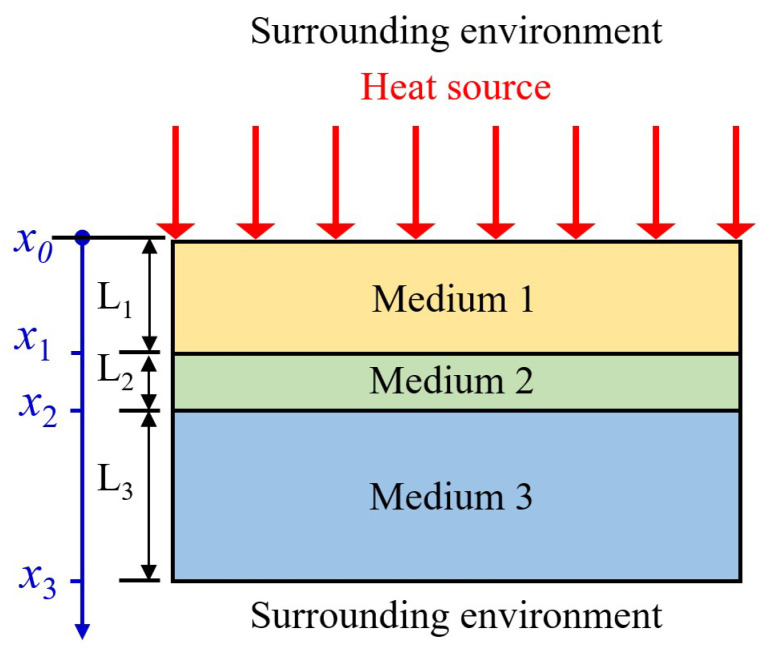
Schematic representation of the one-dimensional model.

An initial analysis of the thermal response was conducted using a one-dimensional (1D) analytical model. This simplified model, which neglects lateral heat diffusion, provides fundamental physical insight into the relationship between excitation frequency and phase response, serves as a benchmark for validating the 3D finite element (FE) simulations in Section 2.3, and establishes a theoretical baseline representing a defect with an infinitely large lateral dimension for quantifying the effects of lateral heat conduction. As shown in Figure 1, a one-dimensional model was built for the thermal response of solid structures containing internal defects, which can provide physical insight for the lock-in thermography. The structure consists of three layers of media, separated by planar interfaces, denoted by *x*_1_ and *x*_2_. The properties of the media are defined as *k*_i_ for thermal conductivity, ρ_i_ for density, *c*_i_ for specific heat, and $\alpha_{i}=\frac{k_{i}}{\rho_{i}c_{i}}$ for thermal diffusivity, where *i* = 1, 2, 3. The sinusoidal heat source is applied on the top surface of Medium 1 and expressed by $\frac{Q_{0}}{2}[1+\cos(\omega t)]$, and ω is the angular frequency. In each layer, the heat conduction equation can be described as

(1) $$\frac{\partial^{2}T}{\partial x^{2}}-\frac{1}{\alpha_{i}}\frac{\partial T}{\partial t}=0\;(x_{i-1}\leq x\leq x_{i},i=1,2,3)$$

1. Boundary condition at the top surface:

(2) −k1∂T∂xx=0+=Q02[1+cos(ωt)]−hf(Tf−T∞)=ReQ02[1+exp(jωt)]−hf(Tf−T∞)

2. Interface conditions between two media:

(3) $$-k_{i}{\left.\frac{\partial T}{\partial x}\right|}_{x=x_{i}^{-}}=-k_{i+1}{\left.\frac{\partial T}{\partial x}\right|}_{x=x_{i}^{+}}(i=1,2)$$

(4) $${\left.T\right|}_{x=x_{i}^{+}}-{\left.T\right|}_{x=x_{i}^{-}}=R_{i,i+1}k_{i+1}{\left.\frac{\partial T}{\partial x}\right|}_{x=x_{i}^{+}}(i=1,2)$$

where $R_{i,i+1}$ is the thermal contact resistance at *x*_i_ between medium *i* and medium *i* + 1.

3. Boundary condition at the bottom surface:

(5) $$-k_{3}{\left.\frac{\partial T}{\partial x}\right|}_{x=x_{3}^{-}}=h_{r}(T_{r}-T_{\infty })$$

4. The temperature solution in each layer can be expressed as

(6) $$T(x,t)=T_{d}(x)+T_{a}(x)\exp(j\omega t)\;(x_{i-1}\leq x\leq x_{i+1},\;i=1,2,3)$$

where $T_{d}(x)$ and $T_{a}(x)\exp(j\omega t)$ are the DC (direct current) component and AC (alternating current) component, respectively, representing the steady-state and periodic components of the temperature response. The solution of Equation (6) has the form

(7) $$\begin{array}{l} T_{a}(x)=A_{i}\exp(-\sigma_{i}x)+B_{i}\exp(\sigma_{i}x)\;(x_{i-1}\leq x<x_{i+1},\;i=1,2,3)\\ \sigma_{i}=(1+j){\left(\frac{\omega }{2\alpha_{i}}\right)}^{\frac{1}{2}} \end{array}$$

Applying the boundary conditions at top and bottom surfaces and interfaces between media, $A_{i}$ and $B_{i}$ can be obtained from the following equation:

$$\left[\begin{array}{cccccc} k_{1}\sigma_{1}+h_{f} & -k_{1}\sigma_{1}+h_{f} & 0 & 0 & 0 & 0\\ -k_{1}\sigma_{1}\cdot \exp(-\sigma_{1}\cdot x_{1}) & k_{1}\sigma_{1}\cdot \exp(\sigma_{1}\cdot x_{1}) & k_{2}\sigma_{2}\cdot \exp(-\sigma_{2}\cdot x_{1}) & -k_{2}\sigma_{2}\cdot \exp(\sigma_{2}\cdot x_{1}) & 0 & 0\\ -\exp(-\sigma_{1}\cdot x_{1}) & -\exp(\sigma_{1}\cdot x_{1}) & \left(1+k_{2}\sigma_{2}R_{1,2})\cdot \exp(-\sigma_{2}\cdot x_{1}\right) & \left(1-k_{2}\sigma_{2}R_{1,2})\cdot \exp(\sigma_{2}\cdot x_{1}\right) & 0 & 0\\ 0 & 0 & -\exp(-\sigma_{2}\cdot x_{2}) & -\exp(\sigma_{2}\cdot x_{2}) & \left(1+k_{3}\sigma_{3}R_{2,3})\cdot \exp(-\sigma_{3}\cdot x_{2}\right) & \left(1-k_{3}\sigma_{3}R_{2,3})\cdot \exp(\sigma_{3}\cdot x_{2}\right)\\ 0 & 0 & -k_{2}\sigma_{2}\cdot \exp(-\sigma_{2}\cdot x_{2}) & k_{2}\sigma_{2}\cdot \exp(\sigma_{2}\cdot x_{2}) & k_{3}\sigma_{3}\cdot \exp(-\sigma_{3}\cdot x_{2}) & -k_{3}\sigma_{3}\cdot \exp(\sigma_{3}\cdot x_{2})\\ 0 & 0 & 0 & 0 & \left(k_{3}\sigma_{3}-h_{r})\cdot \exp(-\sigma_{3}\cdot x_{3}\right) & \left(-k_{3}\sigma_{3}-h_{r})\cdot \exp(\sigma_{3}\cdot x_{3}\right) \end{array}\right]\left[\begin{array}{c} A_{1}\\ B_{1}\\ A_{2}\\ B_{2}\\ A_{3}\\ B_{3} \end{array}\right]=\left[\begin{array}{c} \frac{Q_{0}}{2}\\ 0\\ 0\\ 0\\ 0\\ 0 \end{array}\right]$$

The AC component and phase of the temperature at the top surface are

(8) $$T_{a1}=A_{1}+B_{1}$$

(9) $$\Phi =\arg(A_{1}+B_{1})$$

Here, $A_{1}+B_{1}$ is also a complex quantity. Φ is the phase angle of $A_{1}+B_{1}$, i.e., the phase difference between the temperature response and heat source.

### 2.2. Phase-Difference-Based Defect Detection Method

Using the one-dimensional three-layer model, the temperature responses of the system under lock-in thermography can be calculated with specific parameters, which neglects the effects of lateral thermal conduction. The temperatures of the defective and defect-free models can be calculated separately by defining the material properties of the middle layer. The phase of the defective model is denoted as $\varphi_{d}$, while the phase of the defect-free model is represented as $\varphi_{free}$, resulting in a phase difference that indicates the phase delay caused by the defects as

(10) $$\Delta \varphi =\varphi_{\mathrm{free}}-\varphi_{d}$$

**Figure 2:**
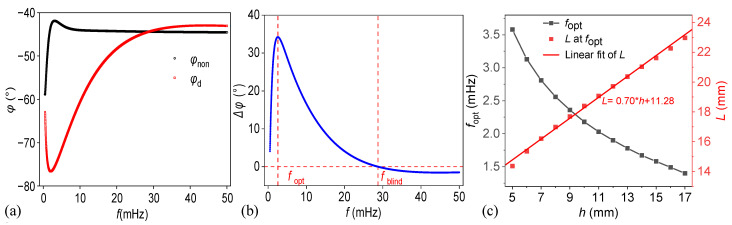
The analytical solutions for the defect (**a**,**b**) at a depth of 8 mm and (**c**) at various depths.

The results were calculated for a titanium alloy model with a total thickness of 30 mm. In the defective model, the material parameters of the second plate were set to air, and all parameters are listed in Table 1. The defective region had a depth of 8 mm (*L*_1_) and a thickness of 4 mm (*L*_2_), and interfacial thermal resistances were zero. Sinusoidal thermal waves with frequencies ranging from 0.5 to 50 mHz, incremented by 0.01 mHz, were used as the excitation heat sources. Specifically, the frequency range was initially estimated based on the sample geometry and the material’s thermal diffusivity, using the relation $\;\mu =\sqrt{\alpha /\left(\pi f\right)},\;$ where *μ* is the thermal diffusion length, *α* is the thermal diffusivity, and *f* is the excitation frequency. The lower bound of the frequency range was selected to ensure that the thermal diffusion length exceeds the specimen thickness, allowing full penetration. In contrast, the upper bound was set so that the thermal diffusion length is less than half the defect depth, enhancing sensitivity to shallow defects. This range enables effective probing of defects across a broad depth spectrum. Figure 2a presents the phase of the temperature response on the top surface of the defective and defect-free models. Figure 2b depicts how the phase difference changes with frequency. In this figure, the optimal excitation frequency is 2.56 mHz with a maximum phase difference of 34.2°, and the blind frequency is 28.74 mHz. Figure 2c shows the variation of the optimal frequency *f*_opt_ (black squares, left axis) and the corresponding thermal diffusion length *L* (red squares, right axis) at the optimal frequency as functions of the defect depth *h*. As *h* increases from 5 mm to 17 mm, *f*_opt_ decreases nonlinearly, while *L* increases approximately linearly. A linear fit of *L* with respect to *h* yields the empirical relationship *L* = 0.70 h + 11.28, as indicated by the red line. This provides an approximate and straightforward method for estimating the optimal excitation frequency based on the relationship between defect depth and thermal diffusion length.

### 2.3. Finite Element Simulations for Thermal Response

**Figure 3:**
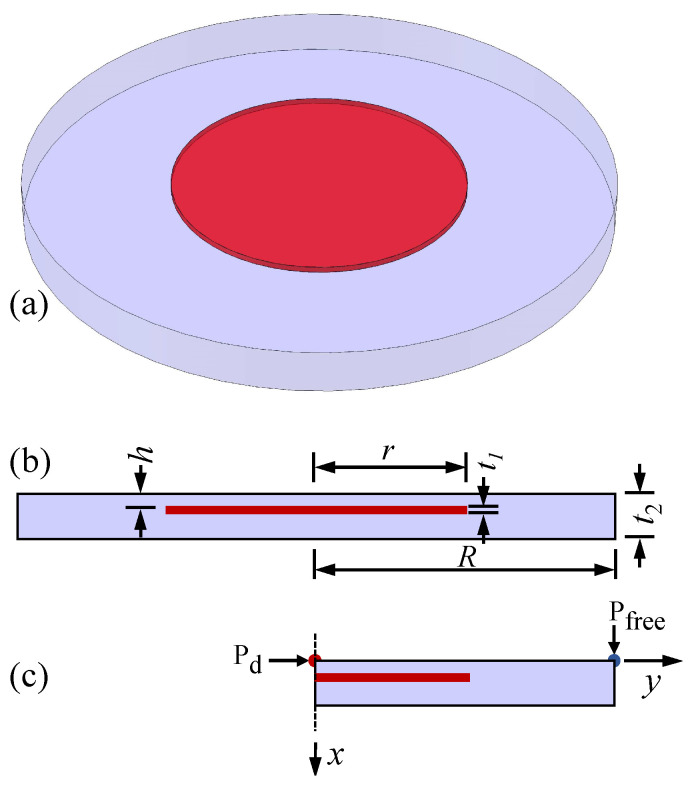
(**a**) Isometric view, (**b**) front view, and (**c**) a simplified axisymmetric representation of the model, with the defect highlighted in red.

A three-dimensional finite element model was developed to evaluate the factors influencing defect detection using the phase difference method. Figure 3a presents the geometric model of a plate featuring a central void defect, which is highlighted in red. Both the plate and defect have a cylindrical shape. The plate is characterized by a radius of *R* and a thickness of $t_{2}$, while the internal defect has a radius of *r*, a thickness of $t_{1}$, and a depth of *h*, as illustrated in Figure 3b. In this study, models with varying defect depths, thicknesses, and radii were developed to investigate how these parameters, along with the radius-to-depth ratio, influence lateral defect detection effectiveness. The specific parameter values used in the simulations are summarized in Table 2 and Table 3, which are organized by model size and grouped according to the varied parameter. Modulated thermal waves were applied to the models, and the temporal temperature responses were recorded at nodes on the top surface of each model. Once the models reached a steady state, the temperature responses from the final cycle were extracted. These data were then analyzed using the Discrete Fourier Transform (DFT) to determine the amplitude and phase at each frequency in the steady-state temperature responses.

Finite element simulations were conducted using ABAQUS-2020. Given the axisymmetry of the model, axisymmetric finite element models were employed for high computational efficiency, as shown in Figure 3c. The vertical dashed line indicates the axis of symmetry. For the thermal response analysis, the DCAX4 element type was adopted. This is a four-node linear axisymmetric quadrilateral element designed for heat transfer applications. All models were discretized with a mesh size of 0.5 mm. A mesh sensitivity analysis was conducted using element sizes of 0.5 mm, 0.375 mm, and 0.3 mm along the thickness direction. The results confirmed that the solution is sufficiently converged with an element size of 0.5 mm. The temperature response at the upper right corner, P_free_, was taken for the defect-free temperature, while the point at the upper left corner, P_d_, locates the center of the defect and is taken to evaluate the temperature variance due to the defects. The material of defect-free regions was set as titanium alloy in the model, while the defective regions were modeled as air. The parameters of material properties are listed in Table 1.

**Figure 4:**
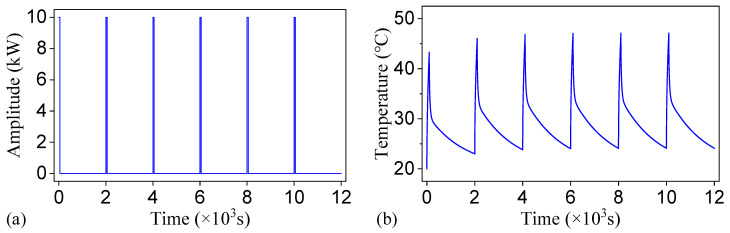
(**a**) Thermal wave heat source and (**b**) temperature response of the defect-free point P_free_.

The initial temperature of the model was set to 20 °C. Convective boundary conditions were applied to both the top and bottom surfaces, with the convective heat transfer coefficient set to 30 W/(m^2^⋅K). A low-duty-cycle square thermal wave method was employed to measure the phase delay on the model’s top surface at the fundamental and harmonic frequencies of the square-wave thermal excitation. In detail, the thermal wave, characterized by a 5% duty cycle, a period of 2000 s (corresponding to a frequency of 0.5 mHz), and a power of 10 kW, was applied to the model. The square-wave thermal excitation with a low duty cycle of 5% was employed to enhance inspection efficiency. This type of excitation signal contains abundant harmonic components in the frequency domain, enabling the extraction of phase and amplitude information across multiple harmonic frequencies from a single thermal response via Fourier analysis. This square wave contains harmonics at multiple frequencies, specifically, integer multiples of the fundamental frequency and the harmonic amplitude is zero only at frequencies that are integer multiples of 20 times the fundamental frequency. Consequently, thermography results can be obtained at both the fundamental frequency and its harmonics. This approach eliminates the need for repeated simulations under varying heating condition. To ensure an adequate signal-to-noise ratio, particularly at higher-order harmonics where signal strength is inherently weaker, the heating power was set to a relatively high level of 10 kW. A fundamental frequency of 0.5 mHz was selected, with its harmonics spanning a broad and relevant frequency range for defect detection. This configuration facilitates a systematic evaluation of the influence of frequency on detection performance, enabling the precise identification of critical information such as the optimal excitation frequency and potential blind frequencies. To ensure that the thermal response had reached a periodic steady-state and to eliminate interference from the initial transient temperature rise, six thermal cycles were applied with the increment size fixed at 0.2 s throughout the simulation, and only the data from the final cycle were utilized in the analysis. Figure 4 shows the thermal wave heat source and temperature response of the point P_free_ at the upper right corner on the top surface.

## 3. Results and Discussion

In this section, the influence of lateral heat conduction, induced by thermal excitation frequency, defect radius, depth, thickness, and the radius-to-depth ratio, on the phase difference at the top surface was analyzed, and the impacts of these parameters on defect detection were assessed. Section 3.1 examines how boundary conditions affect the top surface phase response and detection capability. Section 3.2 examined the lateral heat conduction effect, and demonstrated its significance on the lateral boundary detection. Section 3.3 investigated the impact of defect depth and thickness on phase difference, providing insights into how these geometric parameters affect thermal signal responses. Section 3.4 explored the effect of lateral defect dimension. Section 3.5 examined the radius-to-depth ratio effect, revealing its significance in characterizing defects and limiting detection accuracy. Section 3.6 outlined the limitations of the current study and proposed directions for future research. These analyses collectively demonstrated how phase difference on the top surface can serve as a critical parameter for identifying subsurface defects and quantifying the lateral defect boundary.

### 3.1. Effect of Convective and Radiative Boundary Conditions on Phase Response

**Figure 5:**
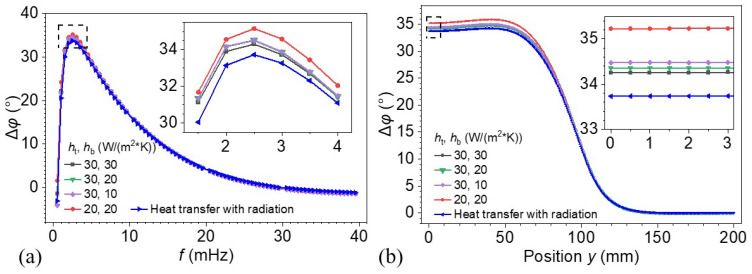
Effects of boundary conditions on phase difference: (**a**) as a function of excitation frequency and (**b**) along different positions on the top surface.

To investigate the influence of thermal boundary conditions on the phase response, a parametric study was conducted by varying the convective heat transfer coefficients on the top (*h*_t_) and bottom (*h*_b_) surfaces, as well as by incorporating radiative heat transfer. Figure 5a presents the phase contrast Δφ at the defect center as a function of excitation frequency under different boundary conditions. The phase contrast values are found to be nearly identical across most excitation frequencies, except for minor differences near the peak, indicating minimal sensitivity to the variation in boundary conditions. Specifically, reducing the convection coefficients at the bottom surface (with *h*_t_ = 30 W/(m^2^⋅K) and *h*_b_ decreasing from 30 to 20 W/(m^2^⋅K)) results in a slightly higher peak phase response. As *h*_b_ is further reduced to 10 W/(m^2^⋅K), the peak phase contrast tends to converge. In contrast, the influence of convection coefficient at the top surface on peak phase response is greater than that of the bottom surface. This is evident from the inset in Figure 5a, where the red curve (representing a lower *h*_t_) shows a noticeably larger peak phase contrast compared to the purple curve. In general, stronger convection slightly reduces the contrast, primarily due to increased surface heat loss, which weakens the thermal gradients induced by subsurface defects. The inclusion of radiative heat transfer further decreases the phase contrast across the optimal excitation frequency range (2~4 mHz), as indicated by the blue curve.

Figure 5b shows the spatial distribution of Δφ along the radial direction on the top surface with various boundary conditions. The region from 0 to 100 mm corresponds to the subsurface defect area, while the region beyond 100 mm represents the defect-free area. Across all cases, a clear drop in phase contrast is observed over the defect region, indicating the sensitivity of the phase signal to subsurface voids. The general shape and position of the phase drop are consistent across all boundary conditions, suggesting robust defect localization. Small differences in the phase contrasts (before y = 100 mm) can be observed, with lower convection and radiation leading to slightly lower values. The inset highlights this effect in the range y = 0~3 mm, where the red curve (20, 20) shows the highest phase contrast, and the blue curve (with radiation) the lowest. This agrees with the result in Figure 5a. Although the convection and radiation boundary conditions slightly influence the magnitude of the phase contrast, they do not affect the shape or location of the defect-induced signal drop. This suggests that the defect detection method is robust to reasonable variations in surface thermal loss mechanisms. Therefore, convection coefficients of 30 W/(m^2^⋅K)) were applied to both the top and bottom surfaces in the subsequent studies, without considering the effect of radiative heat transfer.

### 3.2. Lateral Heat Conduction Effect on the Defect Boundary Detection

**Figure 6:**
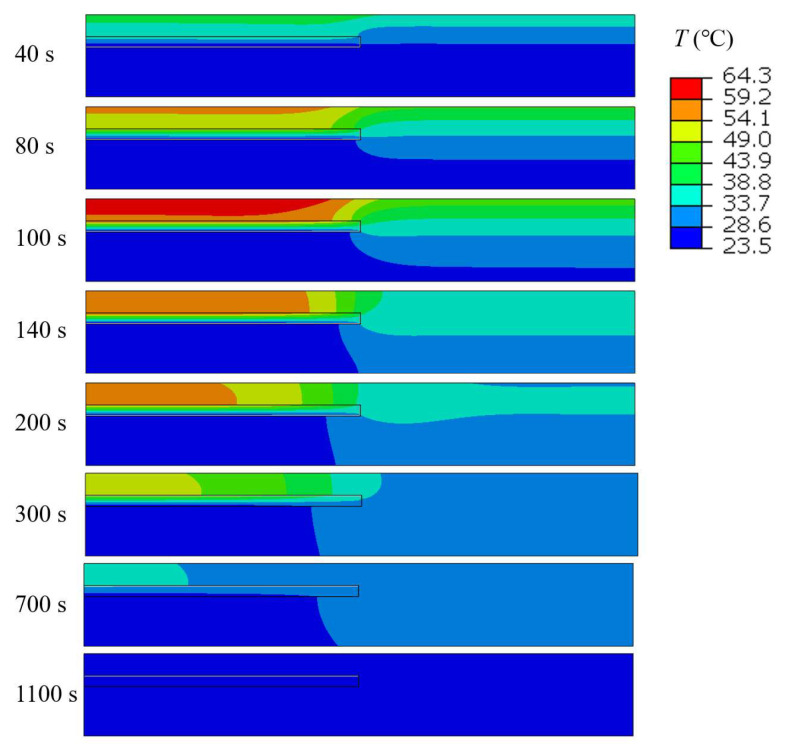
Temperature distribution at different time steps during the final heating cycle.

As illustrated in Figure 3, a model with a radius of 200 mm (*R*) and a thickness of 30 mm ($t_{2}$) was developed, incorporating a defect characterized by a radius of 100 mm (*r*) and a thickness of 4 mm ($t_{1}$), and positioned at a depth of 8 mm (*h*). Figure 6 presents the temperature distribution at selected time intervals (40 s to 1100 s) of the final heating cycle to illustrate the dynamic evolution of heat transfer. The color gradient represents temperature variations, with red indicating the highest temperatures and blue representing the lowest. Although the temperature scale is displayed with a precision of 0.1 °C, this reflects only the post-processing visualization setting in ABAQUS and does not affect the accuracy of the simulation results. The finer resolution helps to illustrate subtle thermal variations more clearly, which is beneficial for defect characterization. Initially, heat penetrates downward from the heated surface and begins to spread laterally. A distinct temperature accumulation is observed above the internal defect around 100 s, due to thermal impedance caused by the void. As time progresses, the temperature field gradually diffuses, and the thermal gradients become less significant. By 1100 s, the system approaches thermal equilibrium, and the defect-induced contrast becomes negligible. These snapshots provide a clear temporal view of the heat propagation process and highlight the critical time window for effective defect detection. Around 100 s, the model shows a significant thermal gradient, with heat primarily concentrated above the defective region. The highest temperatures are observed at the top surface near the defect center, while the temperature decreases gradually as it extends further into the material. The defect, depicted as a rectangular region, disrupts the uniform heat flow, leading to localized thermal variations.

**Figure 7:**
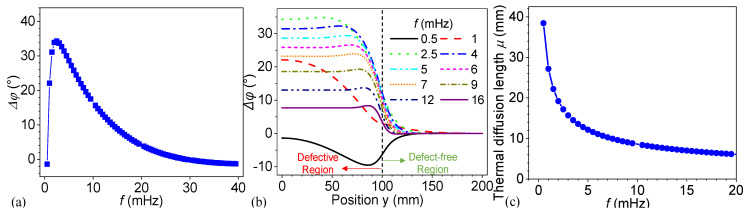
(**a**) Phase difference as a function of excitation frequency, (**b**) phase difference distribution on the top surface at various frequencies, and (**c**) thermal diffusion length in titanium as a function of excitation frequency.

Phase information at harmonic frequencies was obtained by applying a DFT analysis on the temperature signals of all nodes on the top surface. Figure 7a depicts the phase difference between the reference point (P_free_) and the defect center on the top surface (P_d_), as defined in Equation (10), at the fundamental frequency and its harmonics. A positive value indicates the phase delay in the defective region relative to the defect-free region. The curve exhibits a peak phase difference of 34.2° at approximately 2.5 mHz, after which it decreases monotonically with increasing frequency. This peak identifies the optimal excitation frequency of the thermal wave for defect detection with the largest phase difference. The results closely align with the one-dimensional analytical predictions, confirming the reliability of the numerical analysis. The phase difference declines when the frequency exceeds the optimal excitation frequency. At lower frequencies, the system shows a relatively high phase difference, suggesting greater thermal sensitivity in this range. The optimal excitation frequency only confirms the largest phase difference due to the defects.

Figure 7b presents the phase differences between the reference point (P_free_) and the nodes on the top surface as a function of position *y* for various excitation frequencies. The curves represent phase variations across a transition from the left defective region to the right defect-free region, as indicated by the labels. The vertical dashed line at *y* = 100 mm marks the boundary between these two regions. At a frequency of 0.5 mHz, the phase difference exhibits a negative peak within the defective region before transitioning to a near-zero phase difference in the defect-free region. As shown in Figure 7a, 0.5 mHz is much lower than the optimal excitation frequency, leading to an indiscernible phase difference between the defect center and the reference defect-free point. Consistent with Figure 7a, the phase difference between the reference point and the defect center initially rises as the frequency increases, peaks at the optimal excitation frequency shown by the green dotted curve representing the excitation frequency at 2.5 mHz, and then gradually declines. However, the magnitude of the phase contrast at the defect boundary becomes more significant with a larger frequency. Specifically, for curves of 6 mHz and 7 mHz, the phase shift at the defect boundary indicated by the vertical dashed line becomes more abrupt. The reason is that the thermal diffusion length decreases as the frequency increases, as shown in Figure 7c. Thermal diffusion length determines how deeply and widely heat can propagate at a given frequency. Figure 7c illustrates the thermal diffusion length in titanium as a function of excitation frequency. The data reveal a strong inverse relationship: thermal diffusion length decreases sharply with increasing frequency. The longer diffusion lengths at lower frequencies facilitate the detection of subsurface features by allowing thermal waves to penetrate deeper into the material. On the other hand, a lower thermal diffusion length indicates that the lateral heat conduction is restrained. These results suggest that when the thermal excitation frequency starts from the optimal value and increases within a certain range, it enhances phase contrast and thereby improves the detectability of lateral defect boundaries.

**Figure 8:**
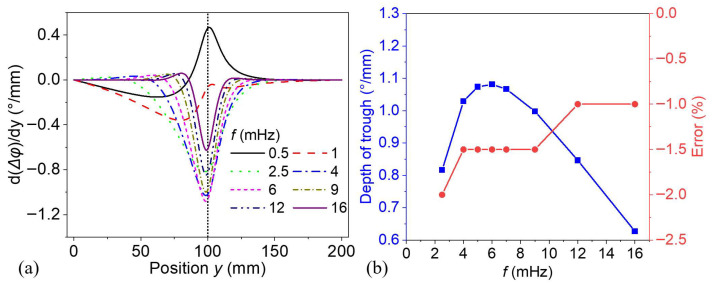
(**a**) Spatial derivative of phase difference curves and (**b**) the depth of trough as well as the error of *y* position predicted by various thermal waves.

Figure 8a illustrates the spatial derivative of phase difference, denoted as d(Δ*φ*)/d*y*, as a function of the surface position *y*, under various thermal wave excitation frequencies ranging from 0.5 to 16 mHz. Each curve corresponds to a specific excitation frequency, where the spatial derivative indicates the rate of change in phase difference across the defect boundary. The pattern of curves is similar when the frequency is above the optimal frequency. The curves show a distinct trough centered near the defect boundary (*y* = 100 mm) marked by the vertical dashed line, indicating a localized phase transition caused by an internal defect. The depth of the trough increases from low frequency (2.5 mHz) and reaches its maximum around 6~7 mHz, then decreases as frequency increases further. This trend, reflected by the blue curve in Figure 8b, suggests that thermal waves ranging from 5 to 7 mHz are more sensitive to the defect, generating the strongest phase gradient response. Deeper troughs imply a sharper phase gradient near the defect edge, caused by stronger interaction between the thermal wave and the defect. At mid-range frequencies, the wave penetrates deep enough to interact significantly with the defect but maintains a high enough resolution to produce a sharp gradient. High frequencies (such as 16 mHz) result in weaker responses with shallower troughs due to limited penetration depth and lower energy reaching the defect.

Thermal waves at lower frequencies have broader troughs as a result of longer thermal diffusion lengths, leading to a wider spread of the thermal response and reduced spatial resolution for defect boundary detection. In contrast, higher frequencies result in narrower troughs, enhancing the ability to pinpoint the defect location precisely. The red curve in Figure 8b illustrates the relative position error, which verifies this statement. Narrow troughs reflect shorter thermal diffusion lengths, allowing for sharper defect edge definition. In all, a balance should be struck by selecting an excitation frequency range that optimizes both boundary sharpness, which aids in distinguishing defective and defect-free regions, and phase contrast, which enhances defect detection.

In Figure 7b and Figure 8, the phase difference curves for 0.5 and 1.0 mHz exhibit distinct behaviors compared to those of higher frequencies. As shown in Figure 7b, neither curve displays a stable phase difference near the defect center (*y* = 0 mm), and they present gradual transitions. In contrast, at higher frequencies, the phase difference remains stable from the defect center to its boundary in the *y* direction, with more localized phase changes at the defect boundary. The defect center is more distinguishable. The length of the stable region increases as frequency rises due to a smaller thermal diffusion length. At lower frequencies, the thermal diffusion length is much larger, which causes the defect boundary’s influence to extend further and affects the phase at the defect center.

**Figure 9:**
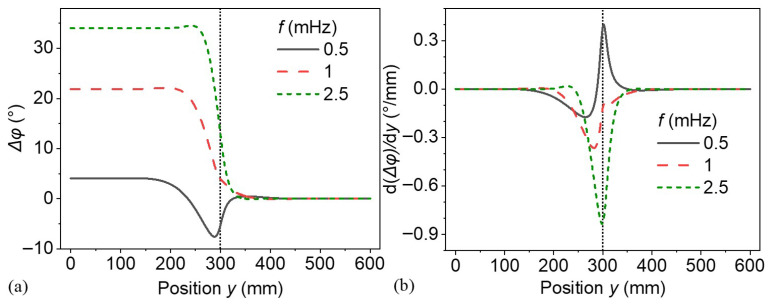
(**a**) Phase differences and (**b**) their spatial derivative on the top surface of a larger model.

To further investigate the phenomenon, a larger model with a radius of 600 mm and a defect radius of 300 mm was constructed, while keeping all other parameters unchanged. The corresponding results are presented in Figure 9. Shown in Figure 9a, as the excitation frequency increases, a distinct trend emerges in the magnitude and spatial range of the phase difference. The lowest frequency (0.5 mHz) exhibits a gradual and wide transition, while the highest frequency (2.5 mHz) shows an abrupt and localized transition in the region centered around 300 mm, indicating the defect boundary by the dashed line. In this expanded model, the phase difference on the top surface under thermal waves higher than 1 mHz exhibits a pattern similar to the curves under frequencies above 2.5 mHz in Figure 7b. The phase difference remains approximately constant from the center of the defect on the top surface up to its boundary. In panel (b), the derivative quantifies the spatial gradient of the phase difference, revealing a sharp peak around y ≈ 300 mm. The gradient is most significant at 2.5 mHz, confirming the steeper spatial transition observed in panel (a). As shown in Figure 7c, the thermal diffusion lengths at 0.5 mHz and 1.0 mHz are 38.43 mm and 27.18 mm, respectively. The longer thermal diffusion length at 0.5 mHz broadens the transition region at the defect boundary. This explains why the phase distribution is more diffused at lower frequencies, whereas higher frequencies result in sharper, more localized phase variations near the defect boundary and apparent defect center. This behavior underscores the sensitivity of the system’s spatial phase characteristics to low-frequency influence.

### 3.3. Defect Depth and Thickness Effect

**Figure 10:**
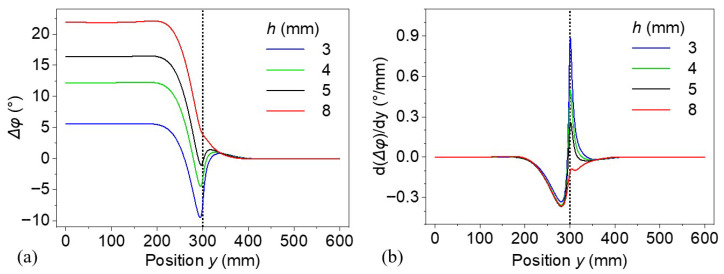
(**a**) Phase differences and (**b**) their spatial derivative on the top surface of models with various defect depths.

Figure 10a shows the lateral distribution of the phase difference along the surface position *y* for defects of varying depths *h* = 3, 4, 5, and 8 mm and a fixed thickness of 4 mm and radius of 300 mm. The radii of these models were set to 600 mm. The excitation frequency of the thermal waves was set to 1 mHz, providing a sufficiently large thermal diffusion length to allow penetration to the defect depth. The phase difference curves reveal an evident dip centered around y ≈ 300 mm, corresponding to the location of the lateral defect boundary. As the defect depth increases, the overall phase difference in defective region becomes larger. The curve corresponding to 8 mm depth has a similar pattern to the curves at frequencies above 2.5 mHz. Figure 10b shows the spatial derivative of the phase difference with the position *y*. The curves for defects with *h* = 3, 4, and 5 mm have similar patterns and show an abrupt change across the defect boundary. The curve for defect depth of 8 mm has a similar pattern to the curves above 2.5 mHz, as shown in Figure 8. Additionally, the position of the trough for *h* = 8 mm shows a slight deviation from the actual defect boundary.

Thermal wave techniques rely on the interaction between modulated surface heating and hidden defect features. The observed phase difference Δ*φ* encodes how much the thermal wave is delayed due to disruptions in the heat flow by the defects. Shallow defects interrupt the thermal diffusion path early, causing sharp phase anomalies near the surface with high spatial resolution. This results in steep phase transitions and strong gradients in the y-direction, as seen for *h* = 3 mm in Figure 10. In contrast, deeper defects allow the thermal wave to diffuse more extensively before encountering a disturbance. This leads to greater overall phase lag (higher phase difference values) due to longer diffusion paths, but the effect becomes more delocalized. Consequently, the thermal wave signature spreads laterally over a wider region, softening the gradient and reducing lateral spatial resolution.

**Figure 11:**
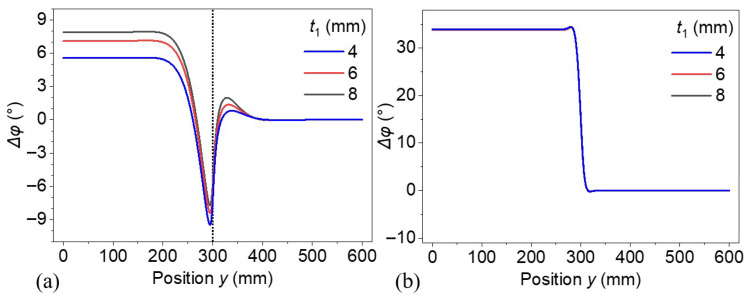
Phase difference distribution for the thermal wave of (**a**) 1 and (**b**) 20 mHz for models with various defect thicknesses.

Models with defect thicknesses of 4, 6, and 8 mm at a fixed depth of 3 mm were constructed to investigate the effect of defect thickness. Figure 11a,b show the lateral distribution of the phase difference on the top surface along position *y* with excitation frequencies of 1 and 20 mHz, respectively. In Figure 11a, the phase profiles exhibit a distinct dip near *y* ≈ 300, corresponding to the location of lateral defect boundaries. As the defect thickness, *t*_1_, increases, the peak phase differences become higher before the dip, and the negative dip becomes shallower. Despite the relative variance in phase difference profiles, the location of defects identified by the derivative of phase difference is less affected by the defect thickness. Figure 11b, in contrast, shows the corresponding phase profile in a different configuration, where all three curves for different *t*_1_ values are virtually overlapping. A sharp, step-like phase transition is centered near the same lateral position, and the insensitivity of the phase response to variations in *t*_1_ implies that the defect thickness has negligible influence under these thermal or structural conditions. The difference in phase profiles shows the effect of thermal diffusion length. The thermal diffusion length at 20 mHz is 6.08 mm, which is much lower than that of 1 mHz (27.18 mm). Hence, the phase response is dominated by the interface condition itself rather than the thickness of defects. This produces a nearly ideal step-like transition in phase, consistent across all thicknesses. These results demonstrate the importance of thermal diffusion length relative to defect thickness in governing the spatial sensitivity of lock-in thermography.

### 3.4. Impact of Lateral Defect Dimensions

**Figure 12:**
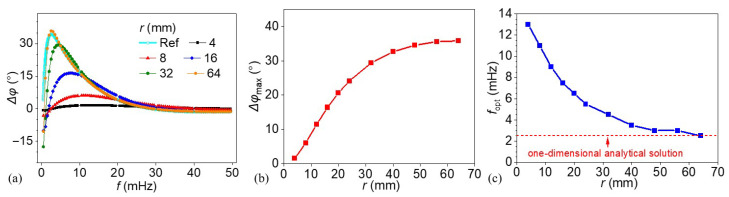
(**a**) Phase difference distributions of the models under different thermal wave excitation frequencies, (**b**) the corresponding maximum phase differences, and (**c**) the optimal excitation frequencies for each defect radius.

The influence of lateral defect dimensions was evaluated by analyzing a series of models with varying defect radii, while maintaining all other parameters constant. In each model, the defects were built with a fixed thickness of 4 mm and a depth of 8 mm, and subjected to an excited thermal wave. The defect radii examined included: 4, 8, 12, 16, 20, 24, 32, 40, 48, 56, and 64 mm. Figure 12 presents a comprehensive analysis of the effect of lateral defect size on the thermal wave response in terms of phase differences. In panel (a), the phase difference Δ*φ* is plotted as a function of the thermal wave excitation frequency *f* for defects with varying radii *r* ranging from 4 mm to 64 mm. Several representative cases are selected and plotted in this panel for clarity. The reference curve (Ref.) represents the analytic solution from the 1D three-layer model, serving as the theoretical baseline for a defect of infinite radius where lateral diffusion is absent. The lateral dimension of defects significantly affects the maximum phase difference and the optimal excitation frequency. A distinct peak is observed in each defect case, with the maximum phase difference shifting leftward (toward lower frequencies) and increasing in magnitude as the defect radius increases. This behavior indicates that larger lateral defects not only amplify the thermal response but also alter the optimal excitation frequency for larger phase contrast. Panel (b) further quantifies the maximum phase difference Δ*φ*_max_ observed for each defect radius. A nonlinear, saturating trend is evident: Δ*φ*_max_ increases rapidly for larger radii but gradually levels off for larger defects beyond 40 mm. This saturation suggests diminishing sensitivity gains at higher defect sizes. Panel (c) presents the inverse relationship between the optimal excitation frequency *f*_opt_ and the defect radius. As the defect radius increases, *f*_opt_ decreases sharply, stabilizing around 2~3 mHz for defects larger than 48 mm. This trend reflects the thermal diffusion required to maximize contrast; lower frequencies (larger thermal diffusion length) are needed for larger lateral defects to achieve maximum thermal interaction and response. Overall, these results emphasize the importance of tuning the excitation frequency based on the expected lateral defect size to maximize detection performance. The saturation behavior in Δ*φ*_max_ and the stabilization of *f*_opt_ at large radii provide practical guidelines for selecting inspection parameters in thermographic nondestructive evaluation techniques.

**Figure 13:**
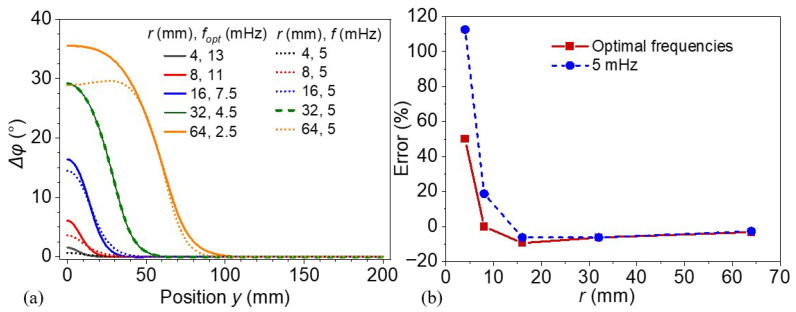
(**a**) Phase difference distributions and (**b**) defect detection error of models with different defect radii under various thermal wave excitation frequencies.

Figure 13 demonstrates the spatial characteristics of the phase differences and quantifies the detection accuracy for defects of various radii under different thermal wave excitation frequencies. Figure 13a illustrates the phase difference distributions (Δ*φ*) across the lateral direction *y* for models with varying defect radii, both under their optimal excitation frequencies (solid lines) and a fixed frequency of 5 mHz (dotted lines). It is evident that under optimal excitation frequency conditions, the phase difference curves exhibit more obvious and localized peaks centered around the defect region. The amplitude of the phase peak increases with defect size, indicating enhanced detectability. Furthermore, larger defects (e.g., *r* of 32 or 64 mm) produce a broader spatial response, while smaller defects show a sharper but narrower phase contrast zone. In contrast, the phase contrast is significantly reduced at the nonoptimized frequency of 5 mHz, leading to poor detectability, particularly for smaller defects.

Figure 13b presents the defect detection error as a function of defect radius under two frequency conditions: individually optimal excitation frequencies (red squares) and the constant 5 mHz (blue circles). The error is defined based on the deviation of the prediction from the defect boundary. A notable finding is that detection errors are dramatically higher for small defects (*r* of 4 and 8 mm) when a fixed frequency is used, which exceeds 100% in the smallest case. In contrast, employing optimal excitation frequencies markedly reduces detection errors across all defect radii, with the most significant improvement observed in the low-radius range. For defects with radii less than 32 mm, the fixed excitation frequency of 5 mHz is considerably lower than the corresponding optimal excitation frequencies, leading to significantly higher detection errors, as discussed in the previous section. For larger defects (*r* > 32 mm), the detection errors obtained using both frequency strategies begin to converge. Notably, for the defect with a radius of 64 mm, the fixed frequency of 5 mHz slightly exceeds the optimal excitation frequency, resulting in a marginally lower detection error. In summary, these results underscore the critical role of excitation frequency in enhancing defect detection using lock-in thermography, particularly for small-scale defects. Employing excitation frequencies reasonably above the optimal value can substantially improve spatial phase contrast and reduce detection errors.

### 3.5. Effect of Radius-to-Depth Ratio

A series of models with varying defect depths (*h* = 8, 10, and 12 mm) were developed to further investigate the influence of defect geometry (specifically the radius-to-depth ratio, *r*/*h*) on detection accuracy. Defect radii (*r*) were set correspondingly, while the defect thickness was maintained at 4 mm. All simulations were conducted under optimal excitation frequencies as listed in Table 4. Table 4 presents the optimal excitation frequencies (*f*_opt_) required for thermal wave imaging across various radius-to-depth ratios (*r*/*h*) and defect depths (*h* = 8, 10, and 12 mm). The data clearly show a systematic decrease in the optimal excitation frequency with increasing *r*/*h* for all depths. This trend reflects the need for lower frequencies to detect larger or more spread-out defects, which require a larger thermal diffusion length due to their broader lateral extent relative to their depth. At a fixed depth of 8, 10, or 12 mm, the optimal excitation frequencies are consistently lower for deeper defects. This is consistent with the principle that deeper defects necessitate longer thermal diffusion lengths, which are achieved using lower excitation frequencies. These results emphasize that both the size and depth of a defect significantly influence the choice of optimal excitation frequency.

**Figure 14:**
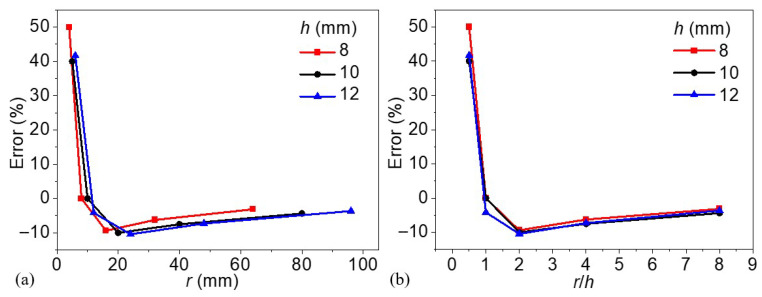
Defect detection errors for models (**a**) as a function of defect radius and (**b**) the radius-to-depth ratio.

In Figure 14a, the detection errors of the lateral defect boundaries are plotted as a function of the absolute defect radius (*r*) with three curves corresponding to models with defect depths of 8, 10, and 12 mm, respectively. A clear nonlinear trend is observed across all depth cases: detection error is highest when the radius is small (i.e., *r* < 10 mm), with values exceeding 40% for all depth levels. This suggests that defects with small lateral dimensions are significantly more challenging to accurately detect and localize, even when the excitation frequency is optimized. As the defect radius increases beyond approximately 16 mm, the detection error rapidly decreases and eventually stabilizes near zero. Notably, the trends across the three depths converge in this regime, indicating that for sufficiently large defects, depth plays a less critical role in influencing detection accuracy once the thermal diffusion length is longer enough, which further verifies the discussion in the above section.

Figure 14b reorganizes the data as a function of the radius-to-depth ratio (*r*/*h*) to better understand the influence of defect geometry. This normalization reveals a strong convergence among all three depth curves, emphasizing the geometric proportionality between radius and depth as a key parameter governing detection accuracy. When *r*/*h* is smaller than 1, detection errors are notably large and positive, indicating significant overestimation or mislocalization of small and deep defects. As *r*/*h* increases beyond 2.0, the detection error approaches a plateau near zero, and the curves for all depth values align closely. This behavior highlights *r*/*h* ≈ 2 as a potential threshold for achieving reliable defect detection of the lateral defect boundaries. In summary, these findings underscore the importance of considering not only absolute dimensions but also geometric ratios when assessing detection performance in lock-in thermography. The convergence of detection errors across depths in the *r*/*h* domain suggests that this parameter can serve as a valuable predictor for optimizing inspection strategies across a range of defect scenarios.

### 3.6. Limitations and Future Work

Despite the insights provided by this study, several limitations should be acknowledged, which could guide promising directions for future research. First, experimental validation was not directly performed in this study due to the technical challenges associated with fabricating well-controlled subsurface defects with precise geometry and depth in metallic materials. Such processes may introduce uncertainties such as geometric deviations or measurement noise that complicate the interpretation of validation results. However, to partially address this limitation, a comparative analysis was carried out using previously published experimental data involving blind holes in metallic plates. The comparison demonstrated good agreement in terms of phase difference [24] and temperature response [28], supporting the credibility of the numerical approach. Future work will focus on conducting controlled experiments using fabricated defect models (e.g., plate with blind holes or coating with embedded inserts) to further validate the simulation-based findings.

Second, the current study assumes prior knowledge of defect geometry (particularly the lateral size and depth) to analyze the influence of lateral heat conduction on optimal excitation frequency and defect detectability. For a given defect depth, the optimal excitation frequency can be estimated using a one-dimensional analytical model, and this frequency increases as the lateral size decreases, offering a theoretical basis for maximizing phase contrast. Moreover, excitation frequencies slightly above the optimal value have been found to provide a good trade-off between phase contrast and boundary resolution. In practical applications, where defect dimensions are typically unknown, a multifrequency or frequency-sweeping strategy may enhance sensitivity to a broad range of defect sizes. It should be noted that the quantitative relationship between defect depth, optimal excitation frequency, and detection performance was not thoroughly investigated in this study and will be addressed in future research.

Third, the analysis is primarily focused on idealized circular defects and homogeneous materials under controlled boundary conditions. Real-world components often involve complex geometries, anisotropic materials, and varying surface conditions. To improve applicability, future work should incorporate more realistic defect shapes, material heterogeneity, and uncertain environmental conditions into both modeling and experimental investigations.

In addition, recent advances in machine learning offer promising avenues for enhancing defect detection. Specifically, sequence-aware neural networks, such as recurrent or transformer-based architectures, are expected to improve defect identification by leveraging the temporal dynamics of thermal wave propagation, which are often overlooked in conventional spatial-phase analyses. Future research will explore the integration of data-driven methods with physical models to develop robust, adaptive defect detection frameworks capable of operating under uncertainty. In summary, these directions may contribute to bridge the gap between theoretical analysis and practical implementation, ultimately improving the reliability and applicability of lock-in thermography for industrial defect detection.

## 4. Conclusions

This study examined several factors influencing the lateral resolution of subsurface defect detection using phase-based lock-in thermography, combining one-dimensional analytical modeling, finite element simulations, and phase analysis. The investigation focused on how thermal diffusion length, governed by excitation frequency, along with defect depth, thickness, lateral size, and the radius-to-depth ratio, affects the detectability and boundary resolution of subsurface flaws. The key findings are summarized as follows:

1. The one-dimensional analytical model provides an initial estimate of the optimal excitation frequency that yields the maximum phase difference for a given defect depth, without accounting for lateral heat conduction. Three-dimensional finite element simulations further demonstrate that the optimal frequency increases as the lateral dimension of the defect decreases, beginning from a value that aligns with the analytical prediction when the lateral dimension is sufficiently large. In contrast, the maximum phase difference decreases with decreasing lateral defect size. The results indicate that lateral heat conduction has a significant impact on the selection of optimal excitation frequency and the accuracy of defect detection.
2. The excitation frequency plays a pivotal role in determining defect visibility and boundary clarity in large-area thermal wave imaging. Lower frequencies are advantageous for detecting deeper defects due to longer thermal diffusion lengths, whereas higher frequencies enhance lateral resolution and boundary localization by restricting lateral heat diffusion. Frequencies below the optimal excitation frequency may result in increased detection error, while frequencies slightly above the optimal value offer a balance between phase contrast and lateral boundary resolution.
3. Both the size and depth of a defect significantly influence the selection of the optimal excitation frequency and the accuracy of defect detection. In contrast, the phase difference is only marginally affected by defect thickness. Shallower defects tend to produce smaller phase differences, making them more difficult to detect. Detecting defects with small lateral dimensions presents a challenge due to reduced phase contrast and spatial resolution. Notably, the geometric ratio between the defect’s radius and depth emerges as a key parameter governing detection performance. The optimal excitation frequency increases as the radius-to-depth ratio decreases, with a ratio of approximately 2 identified as a potential threshold for the reliable detection of lateral defect boundaries. In this way, the study demonstrates that lock-in thermography has limited detection capability for defects with a relatively small radius-to-depth ratio.

Despite the contributions of this study, several limitations remain to be addressed. Future research may focus on the following aspects: experimental validation using controlled blind-hole samples; the development of adaptive multifrequency excitation strategies for detecting unknown defects; advanced modeling of nonideal defect geometries and heterogeneous material conditions; and the integration of temporal deep learning models to improve detection sensitivity and generalizability. These directions are expected to further advance the accuracy and practical effectiveness of thermal wave imaging in industrial applications. Overall, the findings of this study provide important insights and practical guidelines for optimizing excitation strategies and improving subsurface defect detection using lock-in thermography.

## Figures and Tables

**Table 1:** Material properties.

Material	Density (kg/m^3^)	Specific Heat Capacity (J/(kg·K))	Thermal Conductivity (W/(m·K))	Thermal Diffusivity (mm^2^/s)
Air	1.161	1007	0.026	22.24
Titanium	4450	678	7	2.32

**Table 2:** Parameter combinations for simulations of models with R = 200 mm.

Varied Parameter	Defect Depth *h* (mm)	Defect Thickness *t*_1_ (mm)	Defect Radius *r* (mm)
Excitation frequency *f*	8	4	100
Radius *r*	8	4	4, 8, 12, 16, 20, 24, 32, 40, 48, 56, 64
Radius-to-depth ratio *r*/*h*	8	4	4, 8, 16, 32, 64
	10	4	5, 10, 20, 40, 80
	12	4	6, 12, 24, 48 96

**Table 3:** Parameter combinations for simulations of models with R = 600 mm.

Varied Parameter	Defect Depth *h* (mm)	Defect thickness *t*_1_ (mm)	Defect Radius *r* (mm)
Excitation frequency *f*	8	4	300
Depth *h*	3	4	300
	4	4	300
	5	4	300
	8	4	300
Thickness *t*_1_	3	4	300
	3	6	300
	3	8	300

**Table 4:** Optimal excitation frequencies for various radius-to-depth ratios.

*r*/*h*	*f*_opt_ (mHz)		
	*h* = 8 mm	*h* = 10 mm	*h* = 12 mm
0.5	13.0	8.5	6.0
1	11.0	7.0	5.0
2	7.5	5.0	3.5
4	4.5	3.0	2.5
8	2.5	2.0	2.0

## Data Availability

The original contributions presented in this study are included in the article. Further inquiries can be directed to the corresponding author.

## Abbreviations

The following abbreviations are used in this manuscript:

NDT	Nondestructive testing
IRT	Infrared thermography
LIT	Lock-in thermography
